# Supplementary material for: Exercise enhances hippocampal-cortical ripple interactions in the human brain
Source: Brain Commun. 2026 Mar 9;8(2):fcag041. doi: 10.1093/braincomms/fcag041 (PMC13098182; doi:10.1093/braincomms/fcag041)
Supplement: fcag041_Supplementary_Data [file fcag041_Supplementary_Data.docx]

Supplementary Figures

Supplementary Figure 1

Supplementary Figure 1. Single-subject electrode coverage, and hippocampal and cortical ripple detection and co-occurrence at different sites. (A) Single-subject electrode coverage (same as subject in Fig. 3A). Left: Intracortical mesial electrode coverage. Right: Cortical electrode coverage. (B) Raw (<300 Hz), filtered (70-160 Hz) iEEGs, and time-frequency spectral decomposition of the raw iEEG (from the traces marked with asterisk), showing ongoing spontaneous activity from one exemplary hippocampal site (red traces), and one amygdala site (blue traces). Note the spontaneous co-occurrence of high-frequency events (ripples) in both mesial structures. (C) Raw (<300 Hz), filtered (70-160 Hz) iEEGs (from the traces marked with asterisk), and time-frequency spectral decomposition of the raw iEEG (from the traces marked with asterisk), showing ongoing spontaneous activity from several cortical sites of a frontal grid (color coded according to panel A, right). (D) A zoomed-in version of the traces depicted in panel (B), accompanied by its corresponding spectrogram. (E) A zoomed-in version of the traces depicted in panel (C), accompanied by its corresponding spectrogram. (F) Interictal epileptiform discharges (IEDs; marked by black dots) were detected automatically by standard broad-band iEEG (<200 Hz) power threshold-crossing (a 5 SD threshold was used in this case), and confirmed by visual inspection of the iEEG signals. The threshold was selected on the basis of the fact that IEDs are large amplitude and broad-band spectrum events. Contacts with IEDs were excluded from the analysis.

Supplementary Figure 2

Supplementary Figure 2. Pre-processing of intracranial EEG (iEEG) signals using singular value decomposition (SVD) and white matter referencing. (A) Exemplary unprocessed raw iEEG signals (<300 Hz) (no SVD or referencing) in frontal and temporal grid electrodes, and mesial temporal depth electrodes. The traces correspond to the gray-shaded area in Supplementary Fig. 1C. Although common-mode high-frequency noise is ubiquitous, potentially influencing the output of event detection methodologies, physiological ripple activity can still be isolated (red asterisks and right inset). HC: hippocampus; AMY: amygdala. (B) SVD and posterior white-matter referencing eliminate common-mode artifacts from the spontaneous activity shown in (A). Note that ripple activity is more easily trackable by eye (red asterisks and right inset). (C) Band-pass filtered (70-160 Hz) iEEG signals (segment enclosed by the dotted square in panel A, and traces marked with a colored arrow), showing high-frequency artifacts associated with movement and other common sources (black arrows mark prominent noise transients). (D) Analogous to panel C, but displaying the band-pass filtered version of the pre-processed iEEG signals in panel B. Note that high-frequency artifacts are reduced by approximately one order of magnitude. (E) iEEG power (>70 Hz) across 201 hippocampal-cortical pairs (one exemplary subject), showing the overall impact of the pre-processing pipeline on high-frequency activities (**p*=4.65e-18, ranksum test).

Supplementary Figure 3

Supplementary Figure 3. Comparison of referencing methodologies prior to variance-based neural event detection across. (A) Single-subject electrode cortical coverage (same as subject in Fig. 3A and Supplementary Fig. 1) used to schematize referencing methodologies used for comparison to our white-matter bipolar referencing. Common average referencing (CAR) (left schema) subtracts the average across all channels $\bar{\boldsymbol{x}}$ from each channel of interest *x_j_*; whereas in the Gram-Schmidt method (right schema) a modified average $\bar{\boldsymbol{x}}\boldsymbol{'}$ across all channels excluding the channel of interest *x_j_* (depicted in black). The quantity subtracted from *x_j_* is the average $\bar{\boldsymbol{x}}\boldsymbol{'}$ scaled by its similarity to the channel of interest (dots in the formula indicate dot products). (B) Proportion of co-detected neural events via white matter bipolar referencing and CAR across the 7 canonical networks of the Yeo-parcellation, and resting state sessions. Dots indicate individual subjects. Proportion of co-detected events associated with pre- and post-exercise resting state remained non-significant (pre-exercise: VIS – 0.7985±0.0812, SM – 0.8221±0.0663, DA – 0.7949±0.0769, VA – 0.7827±0.048, LIM – 0.7963±0.0737, FP – 0.7980±0.0717, DM – 0.8007±0.0524; post-exercise: VIS – 0.8083±0.0781, SM – 0.7339±0.0821, DA – 0.7261±0.0893, VA – 0.6897±0.0834, LIM – 0.8195±0.0725, FP – 0.7705±0.0728, DM – 0.7658±0.0590; mean proportion of co-detected events with 95% confidence intervals). (C) Similar to panel B, but displaying the proportion of co-detected neural events via white matter bipolar referencing and the Gram-Schmidt method. Proportion of co-detected events associated with pre- and post-exercise resting state remained non-significant (pre-exercise: VIS – 0.7797±0.0875, SM – 0.7530±0.0757, DA – 0.7027±0.0784, VA – 0.7234±0.0740, LIM – 0.8076±0.0763, FP – 0.7724±0.0755, DM – 0.7513±0.0591; post-exercise: VIS – 0.7797±0.0875, SM – 0.7530±0.0757, DA – 0.7027±0.0784, VA – 0.7234±0.0740, LIM – 0.8076±0.0763, FP – 0.7724±0.0755, DM – 0.7513±0.0591; mean proportion of co-detected events with 95% confidence intervals). In box plots, lines crossing the boxes indicate the median, box edges indicate 25th and 75th percentiles, and data points beyond the whiskers (if any) are outliers.

**Supplementary Figure 4**

**Supplementary Figure 4. Ripple detection by non-negative matrix factorization (NMF), and standard band-pass filtering. (A)** Exemplary ripple-band-filtered iEEG trace (70-160 Hz) with overlayed event detection using non-negative matrix factorization (NMF), and standard band-pass filtering. NMF-based detection was implemented on candidate events detected using a broad band filter [10-160 Hz] on the iEEG. **(B)** Exemplary co-detected hippocampal SWR episodes (red dots). Broad band iEEG (5-180 Hz) (top row); ripple-band-filtered iEEG (70-160 Hz) (middle row); and iEEG-associated spectrograms (bottom row). **(C)** Across-subjects co-detection proportion in the hippocampus (HC) (i.e. ripple detection coincidence) between the two event detection methods was 0.8391±0.0547 (mean with 95% confidence interval), thus yielding similar detection results (left subpanel). Dots indicate individual subjects. The null (H_0_) distribution shows the overall coincidence across 500 samples of events with random inter-event intervals with the highest ripple rate across subjects. **(D)** Normalized spectra of individual events from an exemplary recording channel (detection coincidence ~85%), detected by standard band-pass filtering (top) and NMF (bottom). Note that event rates remain consistent and individual spectra remain in good visual agreement. **(E)** Average normalized spectra of the recording channel displayed in panel D across events detected by standard band-pass filtering (*n*=334 ripples; black trace) and NMF (*n*=322 ripples; blue trace). Shadings indicate standard error of the mean (SEM).

**Supplementary Figure 5**

**Supplementary Figure 5. Effect of acute exercise on ripple properties on subjects with bilateral electrode coverage, where only contacts contralateral to the seizure onset zone (SOZ) were analyzed.** **(A)** Across-subjects (*N* = 6) distributions of ripple rate, duration and peak frequency per contact in the resting state pre- and post-exercise in the hippocampus, and **(B)** in the 7 canonical networks, illustrating the modulation of ripple characteristics by acute exercise. In box plots, lines crossing the boxes indicate the median, box edges indicate 25th and 75th percentiles, and data points beyond the whiskers are outliers. As in the main text and Fig. 2, each dot represents one recording site. **p*<0.05, ***p*<0.01 according to a LME model treating subject and recording sites as random effects.
